# Development and Optimization of Tin/Flux Mixture for Direct Tinning and Interfacial Bonding in Aluminum/Steel Bimetallic Compound Casting

**DOI:** 10.3390/ma13245642

**Published:** 2020-12-10

**Authors:** Mohamed Ramadan, Abdulaziz S. Alghamdi, K. M. Hafez, Tayyab Subhani, K. S. Abdel Halim

**Affiliations:** 1 College of Engineering, University of Ha’il, Ha’il P.O. Box 2440, Saudi Arabia; a.alghamdi@uoh.edu.sa (A.S.A.); k.abdulhalem@uoh.edu.sa (K.S.A.H.); 2 Central Metallurgical Research and Development Institute (CMRDI), P.O. Box 87, Helwan 11421, Egypt; khalidhafez@yahoo.com

**Keywords:** nanocomposite, nanoparticle, bimetallic, mechanical, interfacial, compound casting

## Abstract

Interfacial bonding highly affects the quality of bimetallic bearing materials, which primarily depend upon the surface quality of a solid metal substrate in liquid–solid compound casting. In many cases, an intermediate thin metallic layer is deposited on the solid substrate before depositing the liquid metal, which improves the interfacial bonding of the opposing materials. The present work aims to develop and optimize the tinning process of a solid carbon steel substrate after incorporating flux constituents with the tin powder. Five ratios of tin-to-flux—i.e., 1:1, 1:5, 1:10, 1:15, and 1:20—were used for tinning process of carbon steel solid substrate. Furthermore, the effect of volume ratios of liquid Al-based bearing alloy to solid steel substrate were also varied—i.e., 5:1, 6.5:1 and 8.5:1—to optimize the microstructural and mechanical performance, which were evaluated by interfacial microstructural investigation, bonding area determination, hardness and interfacial strength measurements. It was found that a tin-to-flux ratio of 1:10 offered the optimum performance in AlSn12Si4Cu1/steel bimetallic materials, showing a homogenous and continuous interfacial layer structure, while tinned steels using other percentages showed discontinuous and thin layers, as in 1:5 and 1:15, respectively. Furthermore, bimetallic interfacial bonding area and hardness increased by increasing the volume ratio of liquid Al alloy to solid steel substrate. A complete interface bonding area was achieved by using the volume ratio of liquid Al alloy to solid steel substrate of ≥8.5.

## 1. Introduction

Although the development of bimetallic bearings is progressing unceasingly, the stringent requirements of preparing ideal bearings have yet not been achieved. Simultaneously, the demand for outstanding quality bearings is rising continuously with the emergence of advanced technologies in turbines and jet engines. In gas and steam turbines, bearings are used for supporting and positioning the rotating components while journal or roller bearings provide radial support, and axial positioning is acquired by thrust bearings. Ball or roller bearings are generally used for radial support in aircraft jet engines. The desired attributes while designing the bearings are long service life, high reliability, and economic efficiency. Moreover, load, speed, lubrication, temperature, shaft arrangement, mounting/dismounting, noise and environmental conditions are other influencing factors that are considered by design engineers to meet the above specifications.

In most cases, main bearings, such as journal and thrust bearings, are manufactured from bimetallic materials. Bimetallic materials can be fabricated by bonding similar and dissimilar materials. Because of their unique physical, wear and mechanical properties, bimetallic materials were classified as advanced functional materials [1]. The quality of bearings and their lifetime are governed by their wear behavior—i.e., working layer, and bonding strength between the pair metals. The wear behavior of the working layer depends primarily on the bearing material and its structure. The bond between the two metals often depends on their physical, thermal and chemical properties, as well as their fabrication techniques [2,3,4].

Various techniques have been used to fabricate bimetallic bearings, such as casting, welding cast-rolling, cladding and powder metallurgy [1,5,6,7]. Liquid–solid casting (compound casting) is a unique technique for the fabrication of bimetallic bearings, wherein the working bearing materials are in a liquid state (Al-Sn alloy, Sn-Babbitt alloys, and Cu-Sn alloys) and the supported substrate (mild steel, cast iron, copper alloys) are in a solid state. In spite of the development of high-performance materials for bearing applications, there is a continuous demand for increasingly improved tribological properties and higher bonding strength of bimetallic bearing alloys. Although serious efforts have been made worldwide to improve the tribological properties of working layer bimetallic bearing materials, limited work was carried out to improve the bonding strength of the interface layer in bimetallic materials.

In liquid–solid bimetallic castings, metallic interlayers between the two metals are commonly used [6,8,9]. These metallic interlayers are deposited on the solid substrate before pouring the paired liquid metal. The major problems related to the fabrication of bimetallic joints without interlayers are the occurrence of brittle intermetallic phases in the bonding zone that significantly affect the bonding strength of the bimetal elements. In addition, in bimetallic bearings, both metals have different melting temperatures that result in poor wettability between liquid metal and solid substrate [6,8]. Reaction between liquid and solid in liquid–solid casting improves wettability and one of their characteristics is that the wetting angle changes with time as the reaction progresses [10]. If both of the metals of bimetal casting have relatively high melting points, the reaction time will increase, resulting in improved wettability. Otherwise, for low melting point metals, the reaction time will be much lower, resulting in lower wettability. A wise approach is to introduce an additional metal or alloy between the coupled metals and control the structure of the interfacial bonded zone to prevent the formation of intermetallic phases and improve the bimetallic wettability [6,9,10,11]. Recently, Ramadan et al. [9] have improved the shear strength of the Sn interlayer in bimetallic bearings by the addition of Cu. Therefore, Sn, Zn and Sn-Pb alloy are the proposed metals that can be used in the fabrication of interlayers in liquid–solid bimetallic journal and thrust bearings [6,9,11].

Among the available bi-metallic bearing fabrication techniques, liquid–solid static casting technology is considered to be the most economical for bearing layer elements. The improvement of the interfacial bond is a critical factor in the production of bimetallic bearings using a liquid–solid technique wherein the substrate should be considered carefully to achieve higher bond strength, durability and performance. For most liquid–solid bimetallic fabrication techniques, low carbon steel, stainless steel or copper alloys are the nominate materials used as solid substrates [1,9,12,13,14].

It has been reported [11] that Zn interlayer for bimetallic Mg/Al joints significantly increased the bonding strength. Sn and Sn-Pb alloys [6,8,15] are commonly used as interlayers for the fabrication of bimetallic materials using liquid–solid compound casting technique. In our previous work, the Sn metallic interlayer was reinforced by 3 wt.% Cu to improve the bonding strength of Al-Sn/mild steel bimetallic materials [9]. The results showed significant improvement in the shear strength by ~59% compared to the pure Sn interlayer. However, in spite of all the previous works [6,8,9,15] that used the interlayer to improve the bonding strength of bimetallic bearing materials, the bonding strength of bimetallic bearing with interlayer is still low and needs more improvements to increase the performance and lifetime of the bearings.

In view of the above, the current research was designed to develop and optimize a novel tinning process including Sn powder and flux mixture for direct tinning of the solid substrate. The proposed direct tinning process is considered as an alternative new tinning process designed for fast, easy and low-cost surface improvement of solid substrate used in liquid–solid compound casting. This newly designed tinning process is more suitable for flat and horizontal solid substrates like bimetallic thrust bearing applications.

## 2. Materials and Methods

Pure metals, including Sn, Cu, Al and Al-25%Si master alloy, were used for preparing Al-12Sn-4Si-1Cu bearing material. The charge of 1.5 kg of Al-alloy constituents was melted using an electrical furnace in a graphite crucible. Low carbon steel was used for bonding with Al-based bearing alloy to prepare the bimetallic material. The chemical compositions of Al-based bearing alloy and low carbon steel substrate, as manufactured by a local manufacturing Company, Riyadh, Saudi Arabia, are given in Table 1.

**Figure 1:**
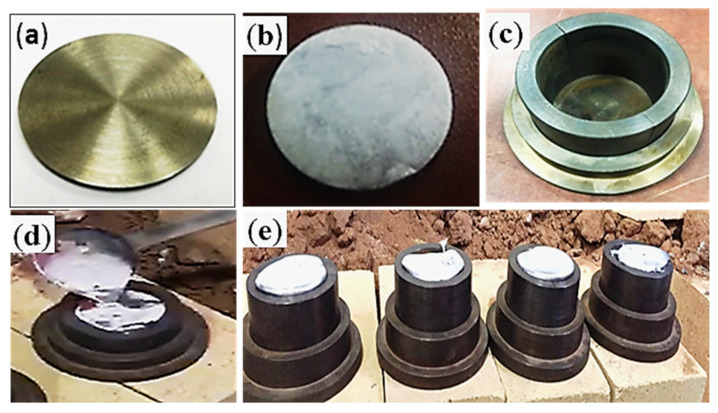
Solid steel substrate (**a**), tinned steel substrate (**b**), metallic mold (**c**), pouring molten Al-based bearing alloy onto tinned steel substrate (**d**), and bimetallic castings after pouring (**e**).

Solid steel cylindrical specimens of 59 mm diameter and 4 mm thickness were grinded with emery papers up to 400 grade. Flux was prepared by mixing zinc chloride, sodium chloride, ammonium chloride and hydrochloric acid in water, as reported in detail in our previous work [9,16]. Five different ratios of tin powder to flux were used for tinning process for bonding liquid Al-based bearing alloy with solid steel substrate—i.e., 1:1, 1:5, 1:10, 1:15, and 1:20. Ratios of tin powder to flux of 1:1 and 1:20 were not characterized due to reasons discussed further below. For a 1:1 ratio, a good distribution of tin/flux mixture on substrate surface was not achieved. For a 1:20 ratio, the amount of Sn was not enough to deposit a considerable Sn-interlayer on the steel substrate surface. For the preparation of bimetallic specimens, the mixture of different ratios of tin and flux were individually spread on a designed area of 2734 mm^2^ of pre-grinded steel specimen. Subsequently the steel specimens with five different ratios of tin/flux mixture were heated on a hot plate for 2 min at 350 °C. Later, steel specimens were washed with warm water and cleaned using cotton cloth to remove the remaining flux from the surface of substrates whereupon the tin layers were produced. Finally, molten Al-based bearing alloy was poured after heating at a temperature of 750 °C into a metallic mold (Figure 1) that contained pre-heated (350 °C) tinned steel specimens.

After solidification, the specimens were removed from the metallic mold for microstructural and mechanical property characterization. For microstructural investigation, the specimens were cut, ground, polished and etched with a solution consisting of 0.5 mL HNO_3_, 0.3 mL HCl, 0.2 mL HF and 19 mL H_2_O. Optical microscope (OM) (Olympus GX51, Tokyo, Japan) and scanning electron microscope (SEM) (FEG-SEM, FEI, Eindhoven, The Netherlands) were used to investigate bimetallic materials and their interfacial structure. The chemical compositions of the bimetallic interfaces were measured by an energy-dispersive X-ray spectroscopy (EDS, Quanta 250 FEG, Eindhoven, The Netherlands).

A Rockwell hardness testing machine was used for measuring the hardness of bimetallic castings. The hardness testing (HRF) was performed at a load of 60 kgf using a 1/16 inch ball indenter for the dwell time of 5 sec. At least five readings of each of the bimetallic specimens were taken for the average value of hardness. The shear strength value of the interface of the bimetallic specimen was performed using a tensile testing machine (Instron 5969, Instron, Norwood, MA, USA), as discussed in a previous work along with the dimensions of the tensile-shear specimen [17]. The dimensions of the tensile shear specimens, having two notches of 4 × 8 × 4 mm on the Al alloy side and of 4 × 8 × 4 on the steel substrate side, were machined to examine the completely exposed bonding interface. The shear test was carried out at a constant strain rate of $\dot{\varepsilon }=3.33\times 10^{-4}\left(\dot{\varepsilon }=V/l_{0}\right)$ and tensile test velocity *V* of 1 mm/min, where *l*_0_ is the distance between shoulders. At least three samples for each Sn-to-flux ratio were used in order to minimize errors.

## 3. Results

**Figure 2:**
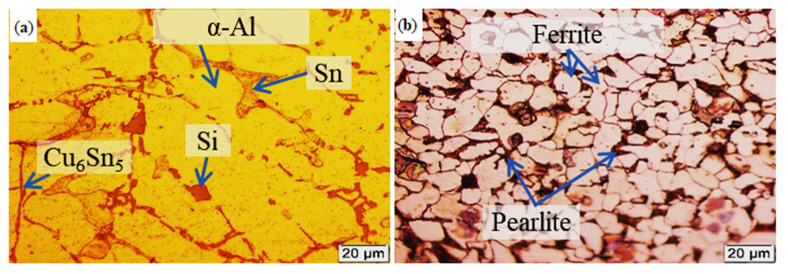
Microstructures of (**a**) Al-based bearing alloy and (**b**) steel substrate used for preparing bimetallic materials.

Microstructures of Al-based bearing alloy and low carbon steel solid substrate used in the fabrication of bimetallic materials are shown in Figure 2. Microstructure of Al-Sn-Si-Cu bearing alloy shows α-Al matrix with Cu_6_Sn_5_, Sn and Si phases (Figure 2a) while the microstructure of low carbon steel has a ferritic–pearlitic microstructure (Figure 2b). The microstructure derives from alloying elements and the treating process parameters, as given in Table 1.

**Figure 3:**
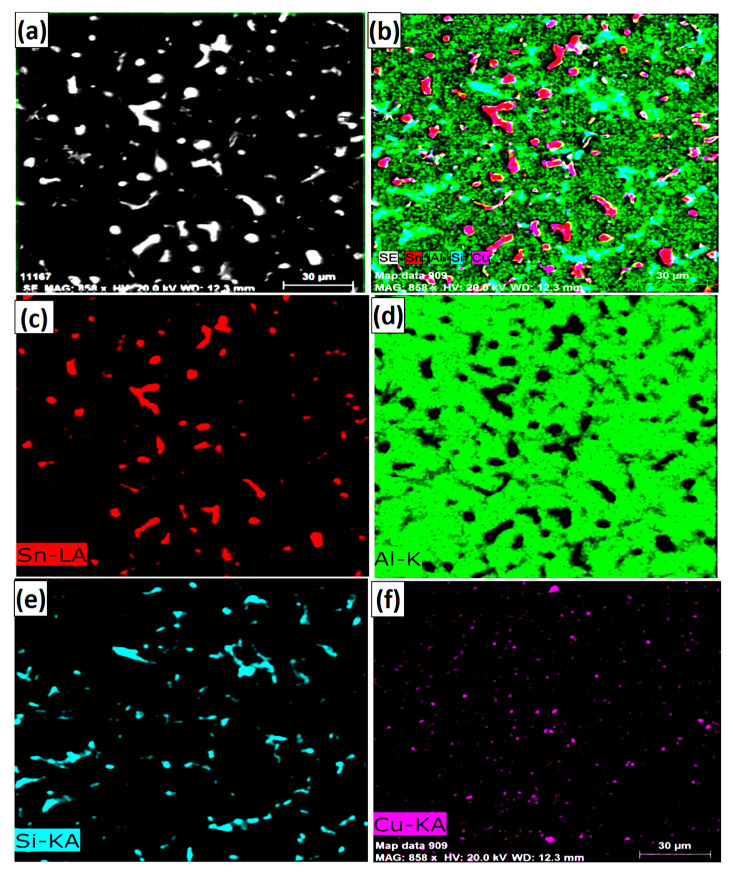
(**a**,**b**) SEM images, (**c**) EDS and (**d**–**f**) mapping analysis of Al-based bearing alloy.

Figure 3 shows SEM microstructures (Figure 3a,b), EDS analysis (Figure 3c) and elemental mapping (Figure 3d–f) of precipitates, phases and matrix of Al-based bearing alloy in bimetallic material. It is clear that the Al-based bearing alloy has fine and well-dispersed precipitates in the α-Al matrix. It was reported [18] that the presence of large particles initiate fatigue, resulting in a low lifetime of Al-based bearing material.

### 3.1. Effect of Sn-to-Flux Percentage on Structure and Properties of Bimetallic Interface

**Figure 4:**
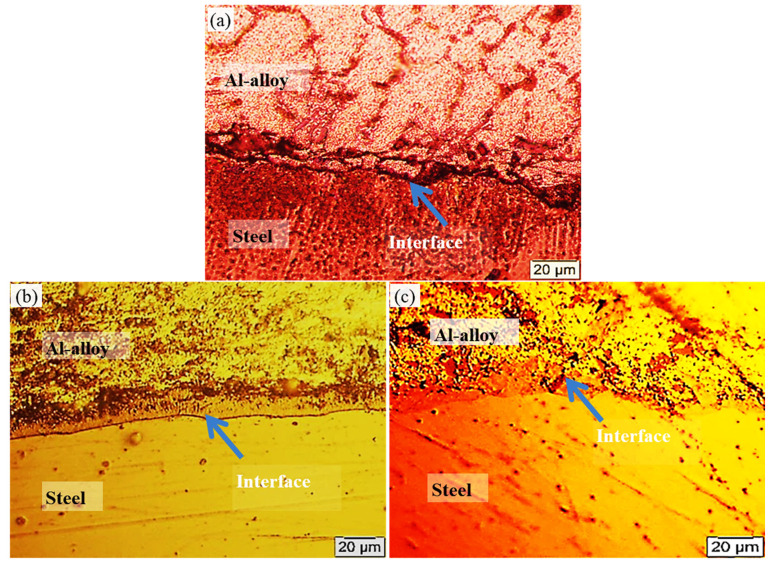
Optical interfacial microstructures of bimetallic materials with tin-to-flux ratios of (**a**) 1:5, (**b**) 1:10 and (**c**) 1:15.

Figure 4 shows the interfacial microstructures of three specimens containing tin-to-flux ratios of 1:5, 1:10 and 1:15. Figure 4a shows an irregular interface wherein the disconnected interface material is also visible at the interface, which may be due to an insufficient flux ratio in the tin/flux mixture (1:5). Using an insufficient flux ratio resulted in decreased wettability of the tin with the steel substrate. The specimen containing 1:10 ratio of tin-to-flux shows a smooth interfacial surface without the formation of isolated regions or areas. In contrast, a continuous layer of tin seems to be developed at the interface. An irregular and relatively thin interface can also be seen in the specimen containing a tin-to-flux ratio of 1:15 but no detachment at the interface was noted in that observed with the specimen of a 1:5 ratio. The development of a continuous tin phase and the emergence of intermetallic phases and/or oxides have their separate effects upon the mechanical performance of the interface, which are discussed further below. 

**Figure 5:**
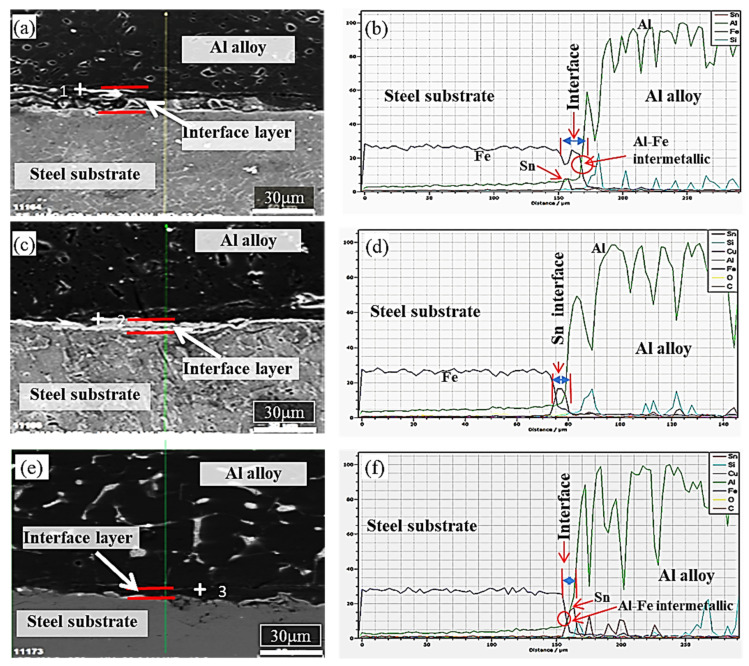
SEM (**a**,**c**,**e**) and EDS (**b**,**d**,**f**) analysis and line scan results across the interface of bimetallic materials using the tin:flux ratios of percentage of 1:5, 1:10 and 1:15.

Figure 5 shows the interfacial SEM microstructures of the three bimetallic specimens containing different tin-to-flux ratios along with the elemental analysis along a line across the interface. The interfacial SEM images (Figure 5a,c,e) replicate the images acquired by optical microscopy and Figure 5a shows the presence of thick interface of specimen containing 1:5 tin: flux ratio indicating the presence of intermetallic phases [19] (Fe-Al intermetallic phases) due to the reaction of aluminum with iron, as revealed in Figure 5b. EDS and line scan confirm the presence of composition of bimetal and the formation of the Sn interlayer. Figure 5c indicates a comparatively continuous and smooth interphase of tin at the interface of adjoining materials of specimen containing 1:10 ratio, which was verified in the graph of EDS line analysis of the same interface indicating the absence of intermetallic phases (Fe-Al intermetallic phases) and the presence of tin at the interface (Figure 5d). Figure 5e shows the interfacial image of the specimen containing a tin-to-flux ratio of 1:15; wherein the thickness of the interphase is discontinuing and relatively thin compared to the previous two samples and the presence of both the tin layer and intermetallic phases (Fe-Al intermetallic phases) can be detected (Figure 5f).

**Figure 6:**
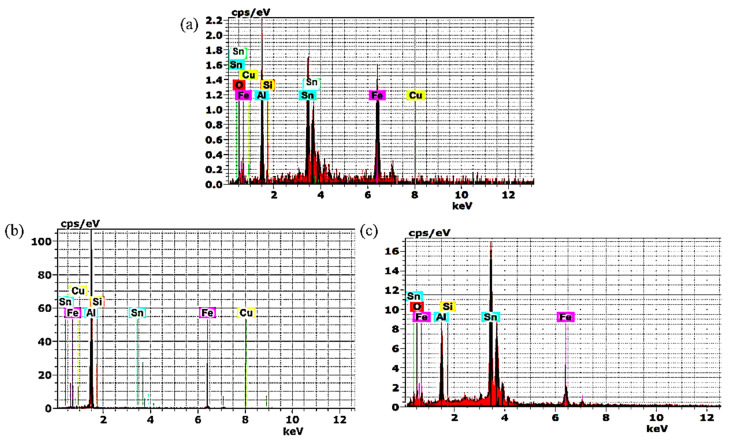
EDS patterns in interface layers adjacent to Al bearing alloy zones in bimetallic castings containing tin-to-flux ratio of (**a**) 1: 5, (**b**) 1:10, and (**c**) and 1:15 at points 1, 2 and 3 shown in Figure 5a–c, respectively.

EDS patterns in interface layers adjacent to Al-bearing alloy zones in bimetallic casting containing a tin-to-flux ratio of 1:5, 1:10 and 1:15 are shown in Figure 6. Higher percentagesa of Fe and Al elements are detected in bimetallic casting containing a tin-to-flux ratio of 1:5 and 1:15 (Figure 6a,c). Figure 6b shows the lowest Fe percentage in bimetal casting containing a tin-to-flux ratio of 1:10. Line scans of Fe and Al intersect across the interface of bimetallic materials (Figure 5a,c) and EDS patterns of points analysis (Table 2) in interface layers adjacent to Al-bearing alloy zones in bimetallic casting (Figure 6a,c), containing a tin-to-flux ratio of 1:5 and 1:15, confirm the formation of Fe-Al intermetallic at the interlayer. Fe-Al intermetallic is, otherwise, absent at the interface of bimetallic casting (Figure 5b) containing tin-to-flux ratio of 1:10 and EDS pattern in interface layers adjacent to the Al-bearing alloy zones in bimetallic casting (Figure 6b) containing a tin-to-flux ratio of 1:10. It shows that the continuous and smooth Sn interlayer can significantly decrease or prevent the formation of Fe-Al intermetallic phases at the interface of Al/Fe. 

**Figure 7:**
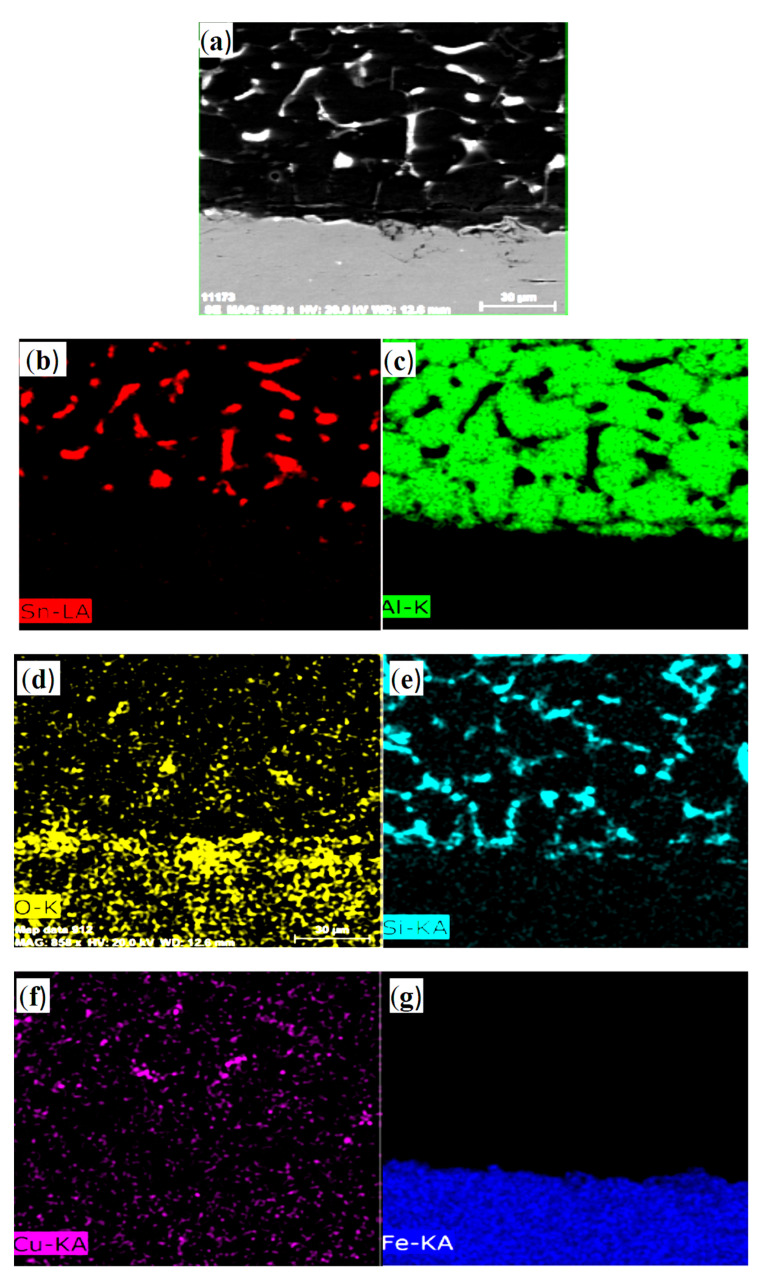
SEM image (**a**), and mapping analysis (**b**–**g**) of bimetallic materials containing a tin:flux ratio of 1:15.

Figure 7a shows the SEM image of bimetallic specimen containing tin-to-flux ratio of 1:15 along with the elemental mapping analysis (Figure 7b–g) of the interface. This specimen was specially chosen for EDS as it contains the mutual effect of the other two specimens containing tin-to-flux ratios of 1:5 and 1:10—i.e., presence of intermetallic phases and the indication of the presence of tin film at the interface. Image in Figure 7b indicates that tin has moved to aluminum and a very low presence of tin was detected in steel. Figure 7c shows the distribution of aluminum in aluminum specimen, while Figure 7g shows the presence of iron in the steel specimen. This shows the absence of migration of aluminum atoms in opposing material while a very low fraction of iron atoms moved to aluminum, as also observed elsewhere [20]. Figure 7d shows the distribution of oxygen across the interface. Comparatively bimetallic casting fabricated using a tin-to-flux ratio of 1:5 has significantly higher oxygen content at the interface, which indicates the presence of tin oxide. The contents of silicon (Figure 7e) in aluminum and steel matches with their relative percentages in specimens, as shown in Table 1. The presence of copper in both the aluminum and steel was also observed (Figure 7f).

**Figure 8:**
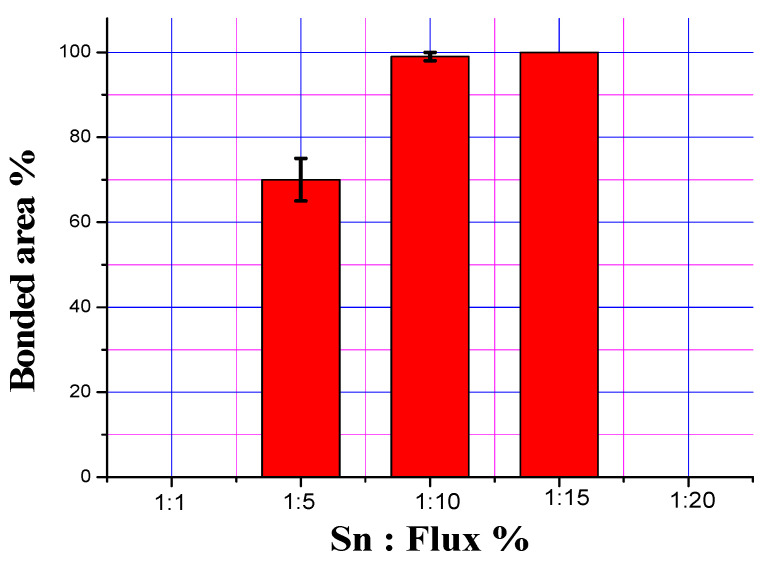
Bimetallic interfacial bonded areas in bimetallic specimens as a function of Sn-to-flux ratios.

Figure 8 shows the interfacial bonded areas of the three bimetallic specimens with tin-to-flux ratios of 1:5, 1:10 and 1:15. It can be seen that a tin-to-flux ratio of 1:5 has the lowest value, while the ratios of 1:10 and 1:15 have shown comparable results: 1:15 ratio has shown comparatively higher value, which is however not significantly higher than 1:10 ratio. The results of bonded areas immediately suggest that the two ratios of 1:10 and 1:15 are better than 1:5 but their quantitative characterization may further explore the preference of one over other, as performed in the interfacial strength measurement test discussed below.

**Figure 9:**
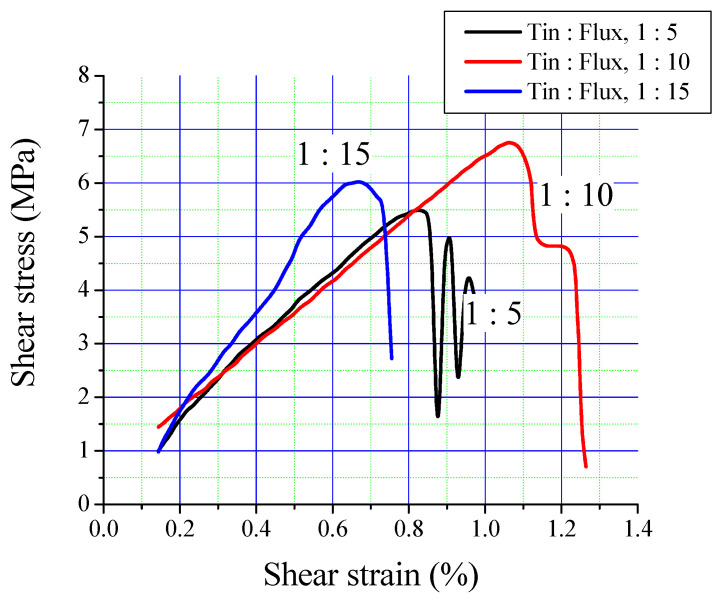
Interfacial shear strength values of bimetallic specimens containing different Sn-to-flux ratios.

Figure 9 shows the stress–strain curves of the three bimetallic specimens with different Sn-to-flux ratios of 1:5, 1:10 and 1:15. Matching with the results of bonded surface areas (Figure 8), the Sn-to-flux ratio of 1:5 demonstrates the minimum stress level (5.5 ± 0.16 MPa), while the stress levels of 1:10 and 1:15 are comparable (6.75 ± 0.30 MPa and 6.0 ± 0.12 MPa, respectively) though 1:10 exhibits a slightly better value. However, the strain value of 1:10 is significantly higher (1.26%) than 1:15 (0.76%), which is still lower than the 1:5 ratio (1.0%). The presence of intermetallic phases in 1:5 and 1:15 may be related to low fracture strain in comparison to the absence of intermetallic phases in 1:10 ratio specimen, as discussed above. In a separate study, Sn + 3%Cu was used along with flux, which significantly improved the shear strength up to 59% in comparison to pure Sn, which was related to the improvement of the interfacial bond structure and low tin oxide content [9]. Although the proposed direct tinning process shows a relatively low shear strength (Table 3), it can be considered as a promising new tinning process for steel for its simplicity, low-cost, easily application and capability to improve by using powder of low melting point solder alloys with a variety of reinforcements.

### 3.2. Effect of Volume Ratio of Liquid-to-Solid on the Interfacial Bonded Area and Hardness

**Figure 10:**
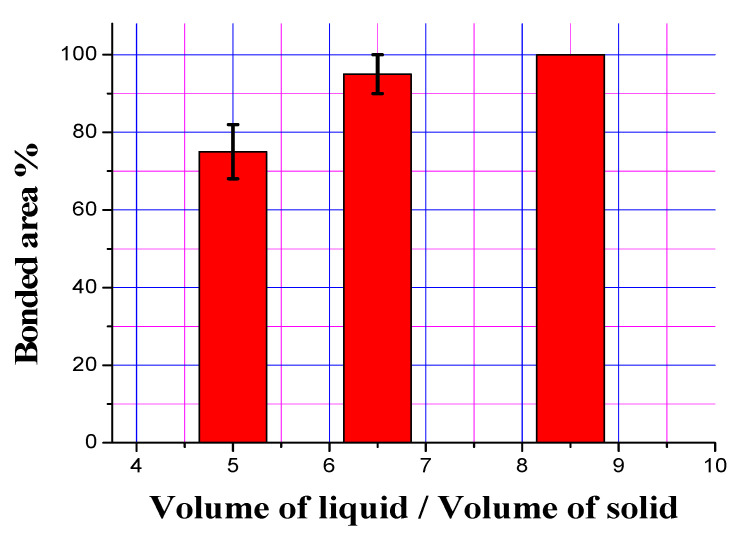
Effect of volume ratio of liquid Al alloy to solid steel substrate on bonded interface area of 1:10 Sn: flux tinned steel substrate.

After optimizing the tin-to-flux ratio of 1:10 in bimetallic specimens of Al-based bearing alloys and carbon steel substrate, the optimization of the ratio of Al-based bearing alloy and carbon steel substrate was also performed (Figure 10). Figure 4b shows interfacial microstructure of bimetal using liquid-solid volume ratio 6.5:1; the other ratios show same interfacial microstructural morphology except that the thickness of interface layer decreases with increasing liquid-solid volume ratio. Three different ratios of aluminum-to-steel were prepared—i.e., 5:1, 6.5:1 and 8.5:1—and it was found that by increasing the content of molten aluminum, which was deposited on the solid substrate and solidified, the bonded area increased. It is to be mentioned here that, in the three aluminum-to-steel ratios, the tin-to-flux ratio was kept constant after optimization. 

**Figure 11:**
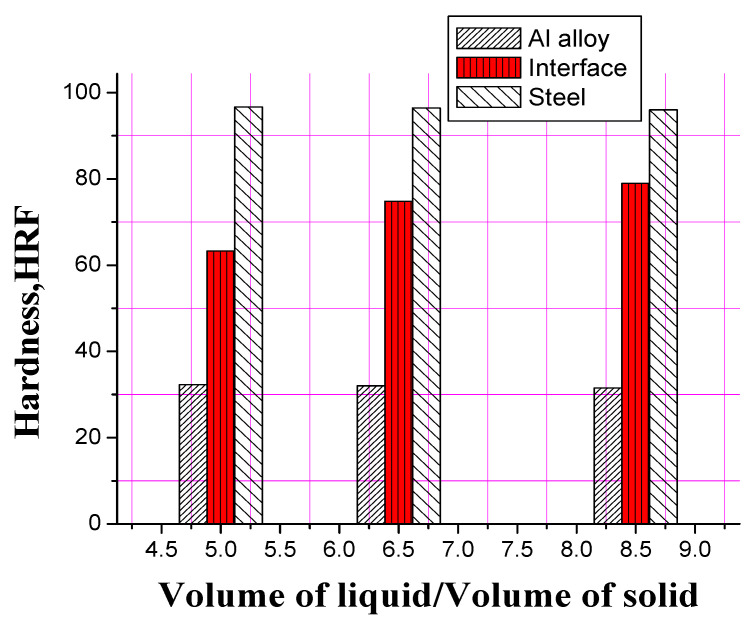
Effect of volume of liquid aluminum to solid steel substrate on interfacial hardness of 1:10 Sn: flux tinned steel substrate.

The hardness characteristics of the three bimetallic specimens containing different ratios of aluminum-to-steel—i.e., 5:1, 6.5:1 and 8.5:1—were measured (Figure 11). Special focus was given on the hardness value determination of the interface, as the hardness of the two opposing materials was found the same in the three specimens. The hardness of the specimens increased continuously with the increase in the bonded area owing to increased molten aluminum deposited on the solid steel substrate.

The achieved results show that the optimum interfacial structure of Al-based bearing alloy/carbon steel bimetal can be accomplished using direct tinning process with tin-to-flux ratio of 1:10. This specified ratio resulted in the formation of a continuous and smooth Sn interlayer between Al bearing alloy and low carbon steel in bimetallic specimens. Fluxes are defined as the chemical compounds that improve the bonding properties of a joint when applied uniformly on the surface to be jointed [23,24]. The 1:5 ratio of tin-to-flux resulted in an irregular interface wherein the disconnected interface material is also visible at the interface. Insufficient flux ratio in tin/flux mixture (1:5) led to the decreased wettability of tin interlayer with steel substrate. Decreasing the wettability of tin with steel substrate results in the improper distribution of tin on the surface of the steel substrate that leads to Al-Fe intermetallic formation at the interlayer between poured Al-alloy and the bare surface of the steel substrate. Otherwise, increased ratio of flux in tin/flux mixture (1:15) leads to decreasing percentage of tin on steel substrate forming a very thin tin interlayer on steel substrate. Before pouring liquid metal (Al-bearing alloy), the tinned steel substrate should be preheated using a hot plate. This preheating process usually partially melts the outer surface of the tin interlayer that leads to a thin layer formation of tin oxide before pouring [25]. In the presence of a thin interlayer of tin, as in the case of 1:15 tin-to-flux ratio, most of the tin interlayer is expected to oxidize. This thin tin oxide layer fails upon the impact of liquid metal pouring onto it due to its brittle nature, as well as its poor wettability with the steel substrate. A fraction of this oxide moves through liquid metal while the remaining stays at the steel substrate (Figure 7b,d).

Aside from the structure of interface and its influence on the properties and strength of bimetallic castings, the desired metallurgical bonding and high-quality bimetallic products cannot be achieved in the presence of unbonded regions and gaps at the interface [4]. It is reported that a good bonding of bimetallic specimen, free of unbonded regions, was fabricated in liquid–solid compound casting using relatively higher volume ratios of liquid to the volume of the solid substrate. In the present study, an Al/Fe bimetal with fully bonded area was achieved by increasing the volume of the liquid to the volume of the solid at 8.5:1. Many metallurgical factors influence a full bond while optimizing the ratio of the volume of the liquid to the volume of the solid in bimetallic specimens. The quantity of the two metals, their melting points, pouring temperatures and interlayer materials are critical factors that determine the optimum ratio of the volume of liquid to volume of solid. Xiong et al. [4] reported that a sound interface between high chromium cast iron and medium carbon steel bimetallic specimen was achieved without using the interlayer but using the liquid–solid volume ratios of ≥10:1. Fathy et al. [6] reported that liquid to solid volume ratio should be kept higher than 5:1 to successfully fabricate Babbitt-steel bimetallic composite at low pouring temperature with the assistance of Sn-Pb interlayer. Similarly, Liu et al. [26] found that the effective interfacial bonding could be achieved using a liquid-to-solid volume ratio of 8:1 that mainly depends on the imported heat energy of the poured liquid metal. One of the previous investigations [4] is in good agreement with the above findings that the liquid-to-solid volume ratios of 5:1 and 6.5:1 are not enough to partially remelt the tin + tin oxide layer (that formed during atmosphere preheating) on the surface of tinned solid steel, resulting in the appearance of an unboned interface area. In contrast, the liquid–solid volume ratio of 8.5:1 imported enough heat energy to achieve optimum interfacial bonding.

## 4. Conclusions

Bimetallic specimens of AlSn12Si4Cu1/carbon steel were successfully prepared by compound casting after depositing the molten aluminum alloy on the solid steel substrate. A novel tinning process comprising the tin powder in combination with varying ratios of flux was performed. Moreover, the optimization of the tinning process was performed after finalizing the tin-to-flux ratio for improved bonded area and corresponding rise in hardness and interfacial shear strength values. The tin-to-flux ratio of 1:10 showed the best combination of interfacial structure, bonded area and interfacial shear strength, which is due to the restriction in the formation of Al-Fe and Fe-Al intermetallic phases due to stable and continuous Sn interface layer formation. Finally, the ratio of the content of aluminum-to-steel was optimized at 8.5:1. Considering it a new process, the direct tinning offers encouraging results in interfacial shear strength characterization, though the scope of further improvement still exists. The developed tinning process, therefore, offers a promising route to manufacture bimetallic flat thrust bearings with improved interfacial strength for hydro, gas and steam turbines, coal pulverizing, and defense applications. 

## Figures and Tables

**Table 1:** Chemical compositions of Al-based bearing alloy and low carbon steel substrate (wt.%).

	C	Si	Mn	Cu	Sn	Cr	Ni	Fe	Al
Al-based bearing alloy	-	4	-	1	12	-	-	-	Bal.
Steel substrate	0.14	0.30	0.48	0.20	-	0.14	0.09	Bal.	-
(Equivalent standard of steel substrate, Japanese Industrial Standards, JIS, G4051)	0.130.18	0.15 0.35	0.30 0.60	≤0.3	-	≤0.2	≤0.2	Bal.	-

**Table 2:** EDS analysis corresponding to the points 1, 2 and 3 indicated in Figure 5.

Number	Element Compositions (at.%)						Inference Adjacent to Al Bearing Alloy, Al, Fe-Al (IMC)
	Al	Fe	Sn	Si	O	Cu	
1	49.6	22.94	14.71	1.16	10.68	0.91	Fe_2_Al_5_
2	94.56	3.71	0.29	1.31	0	0.13	Al
3	30.32	10.44	33.02	0.92	25.31	0	FeAl_3_

**Table 3:** Reports on the shear property of Al/Fe bimetallic composites for different interlay materials and deposition processes in the literature.

Interlayer Material	Shear Stress, MPa		
	Deposition Process		
	Hot Dipping	Electroplating	Direct Tinning
Brass	-	17.5 [21]	-
Al-7.2 wt.% Si	8.5 [22]	-	-
Pure Zn	16.0 [22]	20.0 [17]	-
Pure Sn	-	-	6.75 [This work]
